# Humidity-dependent wound sealing in succulent leaves of *Delosperma cooperi –* An adaptation to seasonal drought stress

**DOI:** 10.3762/bjnano.9.20

**Published:** 2018-01-16

**Authors:** Olga Speck, Mark Schlechtendahl, Florian Borm, Tim Kampowski, Thomas Speck

**Affiliations:** 1 Plant Biomechanics Group, Botanic Garden, Faculty of Biology, University of Freiburg, Schänzlestraße 1, 79104 Freiburg, Germany; 2 Competence Network Biomimetics, Baden-Württemberg, Schänzlestraße 1, 79104 Freiburg, Germany; 3 Freiburg Center for Interactive Materials and Bioinspired Technologies (FIT), University of Freiburg, Georges-Köhler-Allee 105, 79110 Freiburg, Germany,; 4 Freiburg Materials Research Center (FMF), University of Freiburg, Stefan-Meier-Str. 21, 79104 Freiburg, Germany

**Keywords:** hydraulics, mechanical pre-stress, paedomorphosis, self-sealing, wide band tracheids

## Abstract

During evolution, plants evolved various reactions to wounding. Fast wound sealing and subsequent healing represent a selective advantage of particular importance for plants growing in arid habitats. An effective self-sealing function by internal deformation has been found in the succulent leaves of *Delosperma cooperi.* After a transversal incision, the entire leaf bends until the wound is closed. Our results indicate that the underlying sealing principle is a combination of hydraulic shrinking and swelling as the main driving forces and growth-induced mechanical pre-stresses in the tissues. Hydraulic effects were measured in terms of the relative bending angle over 55 minutes under various humidity conditions. The higher the relative air humidity, the lower the bending angle. Negative bending angles were found when a droplet of liquid water was applied to the wound. The statistical analysis revealed highly significant differences of the single main effects such as “humidity conditions in the wound region” and “time after wounding” and their interaction effect. The centripetal arrangement of five tissue layers with various thicknesses and significantly different mechanical properties might play an additional role with regard to mechanically driven effects. Injury disturbs the mechanical equilibrium, with pre-stresses leading to internal deformation until a new equilibrium is reached. In the context of self-sealing by internal deformation, the highly flexible wide-band tracheids, which form a net of vascular bundles, are regarded as paedomorphic tracheids, which are specialised to prevent cell collapse under drought stress and allow for building growth-induced mechanical pre-stresses.

## Introduction

Over the last 3.8 billion years of biological evolution, plants have increasingly evolved diverse mechanisms of wound reactions. High selective pressure on the development of self-repair in the plant kingdom and the independent evolution of various mechanisms of self-repair in the different plant groups and plant species is thus highly probable. During self-repair processes in all the plant species investigated so far, an initial self-sealing phase and subsequent self-healing phase can be discerned. The rapid self-sealing is characterised by a functionally repaired but still present fissure. This initial wound reaction protects the plants from infection by pathogens and may help to inhibit overcritical water loss. These sealing effects give time for the subsequent self-healing of the injury resulting in the disappearance of the fissure, which is structurally repaired in terms of the (partial) restoration of the mechanical properties of the injured organ. Often self-repair is used as an umbrella term comprising both self-sealing and self-healing. Interestingly, these definitions hold true for the self-repair in plants, animals and technical materials, whereas in the latter, the sealing and the healing phase can also be found individually [1].

Self-sealing and self-healing can be characterised by various functional principles, such as physical, chemical and/or biological principles, and can take place on one or more hierarchical levels, namely organs, tissues, cells and extracellular biopolymers [1–5]. In classical materials research, self-healing efficiency is assessed based on the restoration of mechanical properties by a comparison of the change in the function of the intact and healed material [4]. In contrast, in this study, self-sealing efficiency is evaluated via the functional aspect of wound closure. The bending angle is a measure of the degree of wound sealing, whereby total sealing is achieved if the wound surfaces and edges regain contact.

Based on the multitude of biological role models, a variety of bioinspired innovations with self-repair functions have been developed in recent years and more can be expected in the near future [2–3]. Prerequisites for the successful transfer of functional principles into technical applications are the in-depth understanding of the wound reaction of damaged plants and the abstraction of the underlying principles in terms of construction plans, numerical and analytical models [6]. In cases in which mainly physical processes (e.g., movement driven by hydraulics and mechanical instabilities or a given stress–strain field in combination with morphological–anatomical characteristics) and/or chemical processes (e.g., polymerisation, reversible cross-linking) are involved, a transfer to technology is especially promising. However, if biological mechanisms such as metabolism-driven modifications (e.g., biosynthesis, cell proliferation) are present, the technical application is much more difficult. A clear distinction must also be made between cellular and acellular repair mechanisms. In living organisms, cells and their metabolism typically play a major role during the healing processes. They break down old tissues and rebuild new ones. In the context of the development of bioinspired self-repairing materials, we are especially interested in whether metabolic processes occurring in living cells or acellular mechanisms originating in nonliving biological material are involved in the self-repair process and whether one or more hierarchical levels contribute to the self-repair process [1–3].

**Figure 1:**
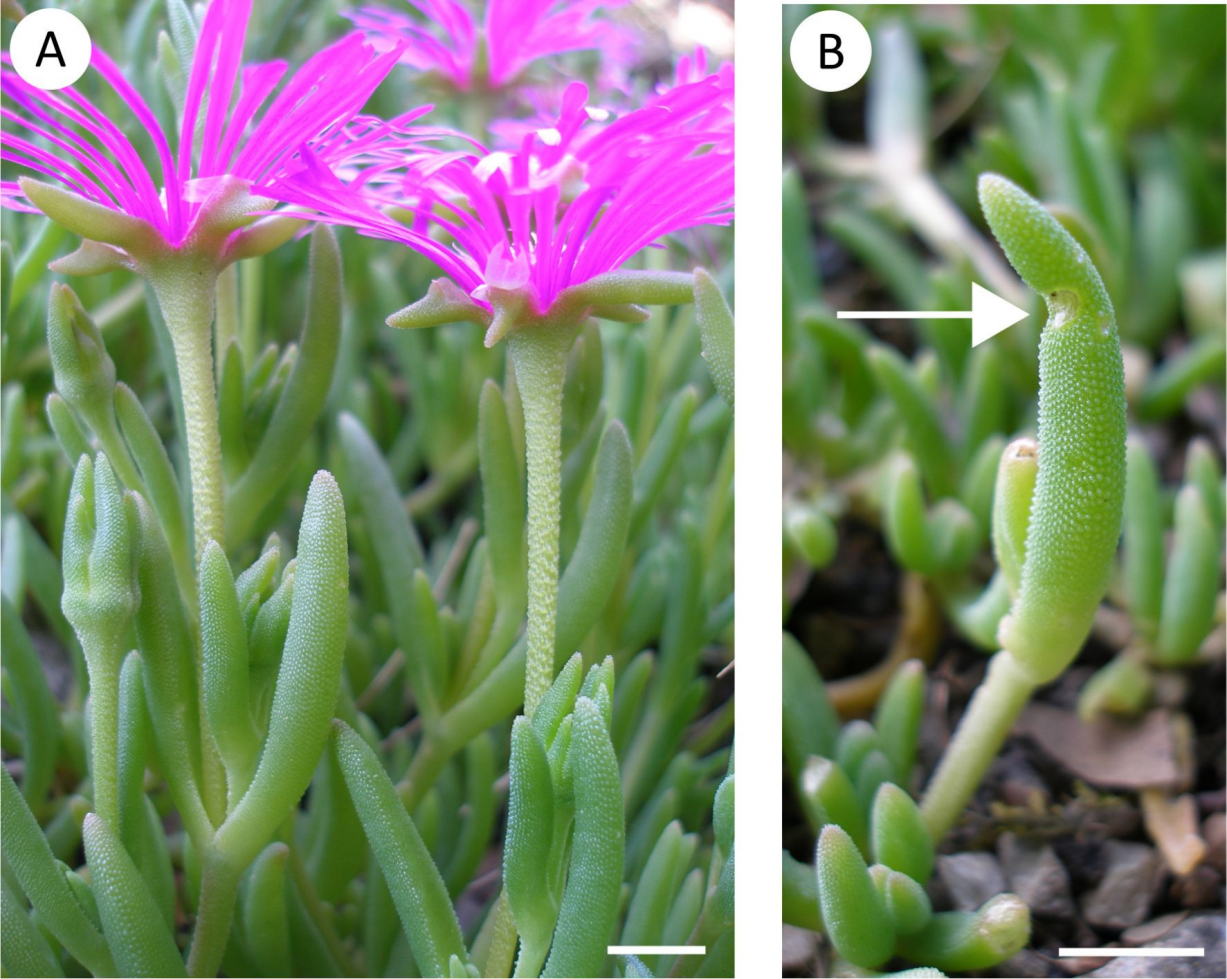
*Delosperma cooperi.* (a) Flowering plants cultivated outdoors in the Botanic Garden Freiburg, Germany. (b) Permanent kink (arrow) after wounding and finalised self-repair of a succulent leaf. Scale bars equal 5 mm.

In the following, we present new findings to further complete the description of the functional principles of self-sealing found in damaged succulent leaves of *Delosperma cooperi* (Figure 1). Initially, the self-sealing phenomenon was described in general and a simplified model was developed to explain the macroscopic movements of the entire plant organ by internal deformations [2–3]. Based on initial anatomical data and mechanical analysis of the leaves, an analytical model was developed that describes the elastic and visco-elastic behaviour of intact leaves and leaves with injuries in the longitudinal, transversal and circumferential direction. This advanced model exclusively takes into account mechanically driven movements and ignores swelling and shrinking effects due to internal water displacement or dehydration [7]. In order to obtain a more detailed and more comprehensive understanding of the underlying functional principles, in the framework of the study presented here, the focus was on the following main aspects. First, with regard to mechanically driven effects, entire leaves and the individual tissue layers composing the leaf have been anatomically and biomechanically characterised in greater detail. Second, to determine hydraulically driven effects, we have carried out analysis of the relative bending angle as a measure of the self-sealing efficiency under various humidity conditions and over time. Third, the obtained results are discussed not only regarding the self-sealing process as such, but also concerning potential ecological aspects and paedomorphosis.

## Results

### Anatomy

**Figure 2:**
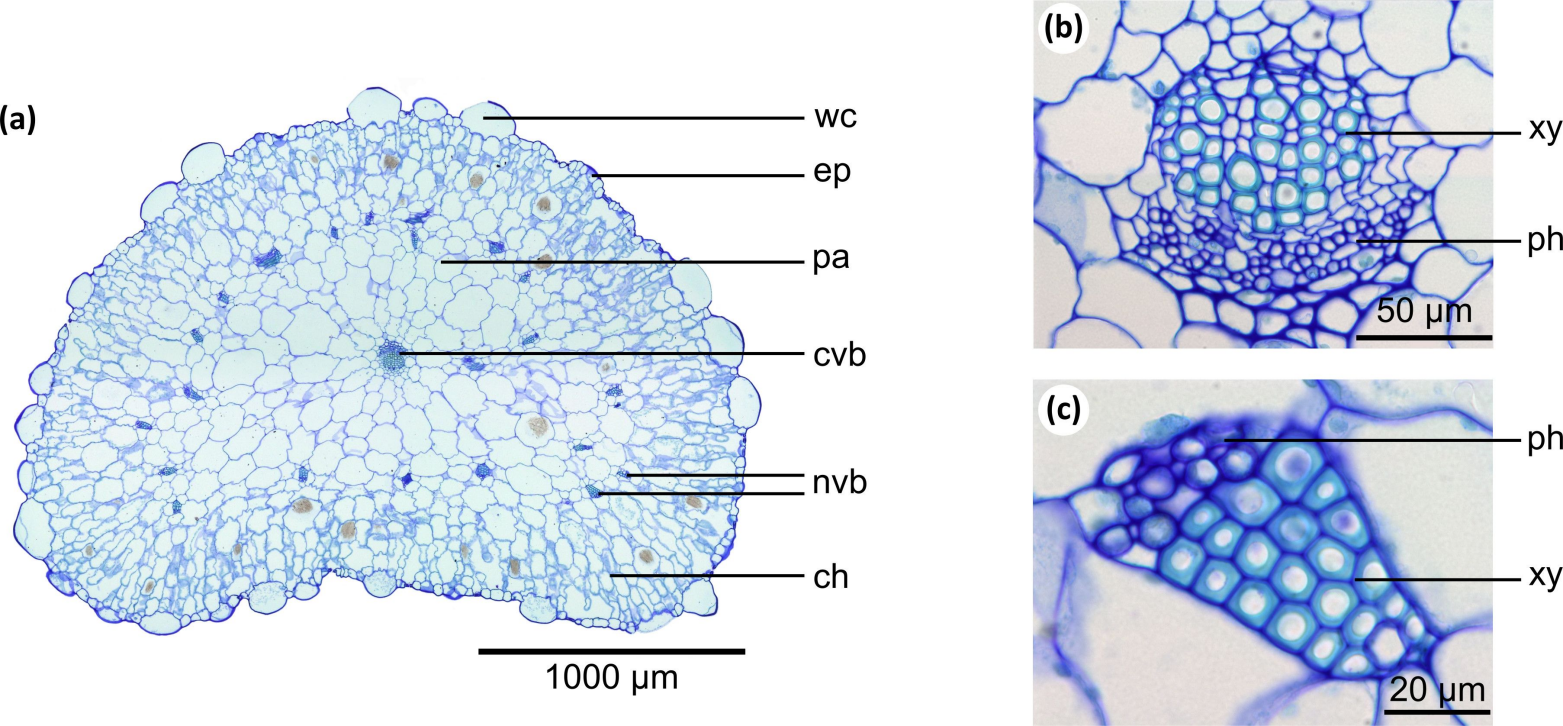
Anatomy of adult leaves of *Delosperma cooperi*. (a) Cross-section of the entire leaf (stained with toluidine blue) comprising five tissue layers: epidermis (ep) with window cells (wc), chlorenchyma (ch), net of peripheral vascular bundles (nvb), parenchyma (pa) and central vascular bundle (cvb). (b, c) Cross-sections of vascular tissues with xylem (xy) and phloem (ph), (b) central vascular bundle, (c) peripheral vascular bundle with wide-band tracheids showing pronounced cell wall thickenings.

Figure 2 shows anatomical details of the succulent leaves of *D. cooperi*. The leaf shape ranges from oval near the base to round at the apex. Cross-sections reveal a centripetal arrangement of five tissue layers, each having a characteristic thickness (Table 1). From the outside to the inside, the following layers can be observed: an epidermis layer with window cells, a peripheral ring of chlorenchyma, a thin net consisting of vascular bundles, an inner ring of parenchyma and a strand of vascular bundles in the leaf centre (Figure 2a).

**Table 1:** Morphometric data of tissue layers found in adult leaves of *Delosperma cooperi.* Measurements derived from thin sections. IQR stands for interquartile range.

Thickness (µm)					Radius (µm)
	Epidermis (without window cells)	Chlorenchyma^a^	Net of vascular bundles^b^	Parenchyma^a^	Central vascular strand

median	31.82	461.78	15.11	590.81	50.92
IQR	3.66	102.29	3.94	119.64	7.88
minimum	26.14	359.75	11.97	497.59	39.45
maxium	38.50	703.30	19.07	814.46	61.26
*n*	10	10	10	10	10

^a^During preparation of the thin sections, succulent parenchymatous tissues have shrunk by approximately 15%. ^b^The net of vascular bundles was converted to a continuous ring having a thickness that was calculated by dividing the total area of all peripheral vascular bundles by the term 2π*r*, with *r* being the middle radius of the net.

In the central vascular strand, most tracheids are scalariform, reticulate or pitted; sporadically, tracheids with annular and helical wall thickenings can also be found (Figure 2b). In contrast, the net of vascular bundles almost exclusively comprises tracheids with annular and helical wall thickenings (Figure 2c). Moreover, the net of vascular tissue consists in part of wide-band tracheids, a specialized type of tracheid that prevents cell collapse under (high) drought stress. Wide-band tracheids are short, wide and spindle-shaped and possess pronounced annular or helical cell wall thickenings. They consist of an unlignified primary cell wall and a band-like secondary cell wall that project deeply into the cell lumen [8–14]. Up to 70% of the cross-section of a tracheid can be filled by these thickenings.

### Biomechanics

Table 2 summarizes the mechanical properties and other mechanically important parameters. Mechanical properties of the entire leaf and of single tissue layers were measured in tensile tests, which provided the basis for calculating the elastic modulus and tensile strength. The elastic modulus of the epidermis in transversal and longitudinal directions was not significantly different (Wilcoxon Mann-Whitney signed rank test, *W* = 38, *p* > 0.05), whereas the tensile strength differed significantly (Wilcoxon Mann-Whitney signed rank test, *W* = 19.5, *p* < 0.05). On the basis of Equation 1 (presented later in this work), we calculated the elastic modulus of the parenchyma and chlorenchyma. On the assumption that, within a species, the parenchyma and chlorenchyma cells did not differ markedly concerning anatomy or turgor, they could be treated jointly in the respective species. By taking into account the median values of the measured parameters such as turgor *P* = 0.042 MPa, cell diameter *d*_c_ = 77 µm, cell wall thickness *t*_cw_ = 0.42 µm and Poisson’s ratio *v* = 0.28 and by using an elastic modulus of cell walls *E*_cw_ = 5.00 MPa [15], an elastic modulus of 0.25 MPa was calculated for the parenchyma and chlorenchyma. Poisson’s ratio from entire leaves was calculated from pictures taken during tensile tests. No correlation could be found (Spearman’s rank-order correlation, Spearman’s *rho* = −0.29) between the turgor of single cells and their position in the leaf. Nevertheless, the turgor tended to decrease slightly with increasing measured depth.

**Table 2:** Mechanical properties and additional mechanically important characterization of adult leaves of *Delosperma cooperi.* IQR stands for interquartile range.

	Median	IQR	Minimum	Maximum	*n*

elastic modulus (MPa)					
leaf	0.72	0.47	0.23	2.14	18
central vascular strand	32.80	23.20	17.71	51.19	8
epidermis (transversal)	3.62	0.65	3.05	5.43	10
epidermis (longitudinal)	5.27	3.42	2.25	8.31	10
parenchyma and chlorenchyma^a^	0.25	0.06	0.20	0.43	44
tensile strength (MPa)					
leaf	0.09	0.03	0.08	0.15	13
central vascular strand	8.80	1.69	5.83	11.27	8
epidermis (transversal)	1.25	0.19	0.88	1.44	10
epidermis (longitudinal)	1.54	0.37	1.10	1.90	10
Poisson’s Ratio					
leaf	0.28	0.14	0.12	0.42	18
turgor (MPa)					
parenchyma and chlorenchyma	0.04	0.02	0.03	0.09	44

^a^Calculated by Equation 1 (presented later in the Experimental section of this article) by using the median of the turgor measurements.

### Self-sealing efficiency

**Figure 3:**
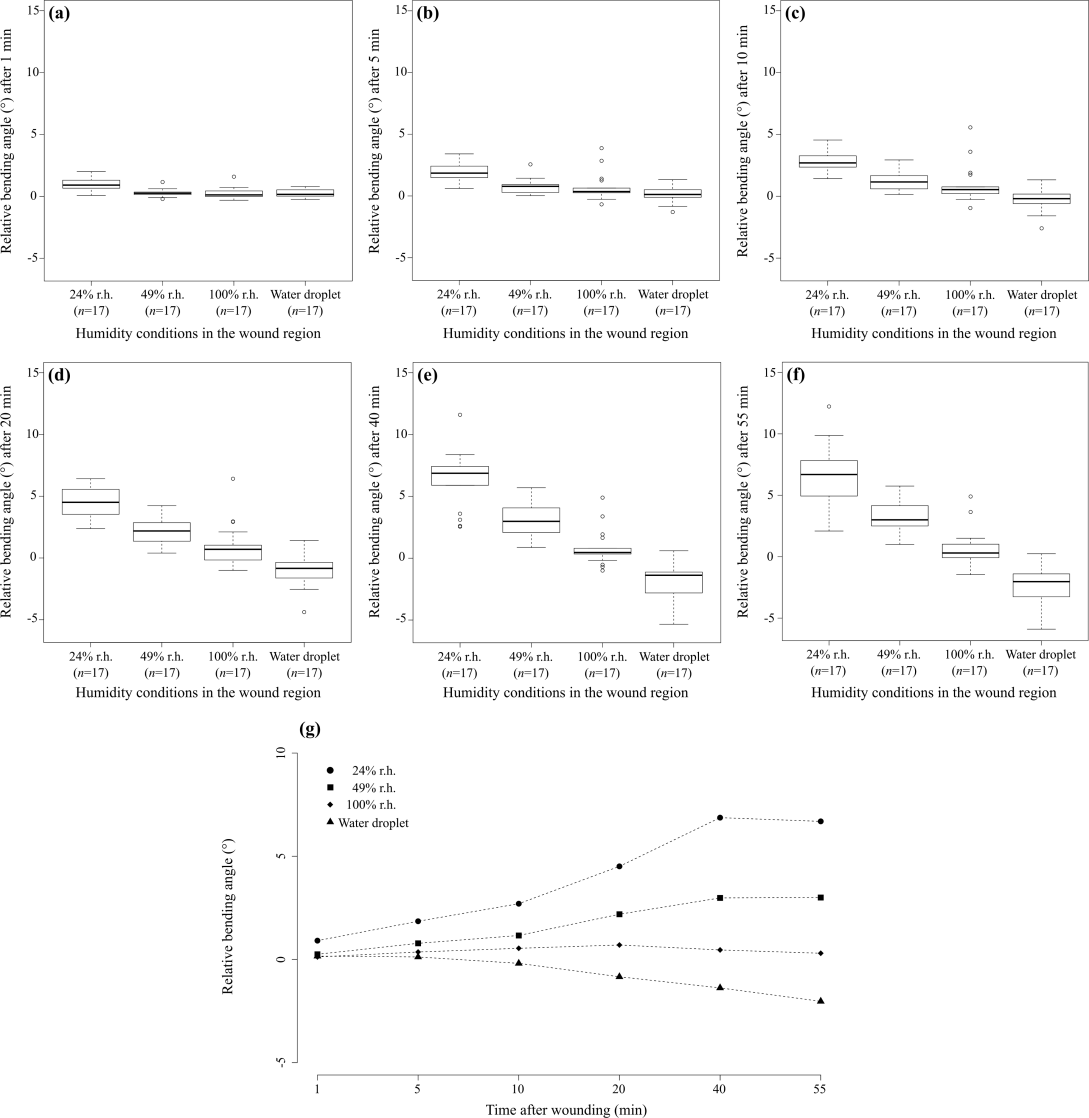
Self-sealing efficiency of *Delosperma cooperi* leaves. Diagrams show the relative bending angle as a function of “humidity conditions in the wound region” (r.h. = relative air humidity and water droplet) and “time after wounding”. (a–f) Comparative box and whisker diagrams display the relative bending angles γ_n_ dependent on “water” in terms of relative air humidity (r.h.) at 24, 49 and 100% and after a water droplet was applied to a transversal wound after (a) 1 min, (b) 5 min, (c) 10 min, (d) 20 min, (e) 40 min and (f) 55 min. (g) For better traceability the significant interaction effect of “humidity conditions in the wound region” and “time after wounding” is presented as the median of measured data (note that the statistical analysis was carried out on rank-transformed data showing the same results).

Figure 3 shows the quantitative analysis of the relative bending angle γ_n_ after a transversal wounding as a measure of the self-sealing efficiency depending on various humidity conditions in the wound region (24, 49 and 100 % relative air humidity, water droplet) (see Supporting Information File 1) and as a measure of the dynamics of the sealing mechanism in terms of time dependence (after 1, 5, 10, 20, 40 and 55 minutes). The results show that, in general, the relative bending angle decreases with increasing air humidity and tends to increase over time after wounding, that is, the wound increasingly closes. In the case of the addition of a water droplet, the observed leaf movement markedly differs as the wound size increases, as is mirrored by the negative values of the relative bending angle (see Supporting Information File 2).

The statistical analysis revealed highly significant differences for the single factors “humidity conditions in the wound region” and “time after wounding” and for their interaction effect (two-way repeated measures ANOVA on rank-transformed data, see Supporting Information File 3; “humidity conditions in the wound region”, *F*_3,48_ = 73.31, *p* < 0.001; “time after wounding”, *F*_5,80_ = 29.86, *p* < 0.001; interaction effect, *F*_15,240_ = 40.51, *p* < 0.001). A pairwise comparison of the various relative air humidity values and the water droplet demonstrated, without exception, highly significant differences (see Supporting Information File 3). The comparisons of the bending angles at various time spans after wounding showed significant differences for pairwise *t*-tests of 1 min with all other time spans and of 5 min with 10 and 20 min (see Supporting Information File 3). For all other pairwise comparisons of “time after wounding”, no significant differences could be found (see Supporting Information File 3).

## Discussion

Ice plants growing in arid or semi-arid habitats of South Africa have evolved a number of adaptations for survival, such as the storage of water (i.e., succulence), the prevention of collapse of the water-conducting elements under drought stress (i.e., wide-band tracheids) and the avoidance of water loss (i.e., self-repair). The aforementioned adaptations are mutually dependent and supportive. *D. cooperi* is native to the cold semi-arid climate of South Africa (Köppen climate classification BSk) where the average annual temperature ranges from 7 to 24 °C, the annual average relative humidity is 57%, and the precipitation is about 500 mm per year with 58 rainy days. In the dry winter month of September, statistically, only 2 rainy days and a relative humidity of 46% occur close to the town of Bloemfontain [16]. In the context of our experiments on self-sealing efficiency, high values of relative humidity can be associated with fog and the liquid water droplets with rain. The fast sealing of injuries in order to prevent dehydration might be an effective adaptation and selective advantage for *D. cooperi* plants growing in such semi-arid habitats. An additional aspect that may increase the selection pressure on an efficient self-sealing mechanism is the finding that, in the dry winter months, birds feed from *D. cooperi* leaves and thereby cause frequent injuries [17]. In addition to other adaptations, the ability to efficiently seal injuries, especially at low relative humidity, may contribute to the remarkable speciation burst of ice plants in South Africa [14].

A more specialised adaptation to high seasonal drought stress is the presence of wide-band tracheids [8–14]. In contrast to common tracheids, they efficiently prevent cell collapse under drought stress. Because of their wide and lignified secondary wall thickenings, wide-band tracheids maintain their shape and function far longer than tracheids without these thickenings and should readily allow rehydration [10]. These specialized tracheids with annular, helical or double-helical secondary wall patterns evolved independently within the families Aizoaceae, Cactaceae and Portulacaceae [8,12]. Wide-band tracheids are often found in seedlings and young plants. They are typically replaced by tracheid types with scalariform and reticulate thickenings, which are more rigid in bending, compression and tension during ontogeny and which are more efficient in supporting the larger adult woody plant body. However, in some species, adult plants remain locked in the production of initial wide-band tracheid in their wood. This can be interpreted as a case of paedomorphosis [12–13]. In leaves of *D. cooperi*, the net of vascular bundles consists almost exclusively of wide-band tracheids. These paedomorphic vascular bundles guarantee a high degree of extensibility and, as a consequence, a higher efficiency of self-sealing by internal deformations. The associated reduction in dehydration of damaged leaves may be a preadaptation and selective advantage for the colonisation of arid and semi-arid habitats. In the context of self-sealing by movement of the entire leaf, highly flexible wide-band tracheids most probably play an especially interesting role.

Because the hydraulically and mechanically driven effects of the self-sealing movement found in *D. cooperi* leaves seem to be closely interwoven, they are jointly discussed. In general, the movement of multicellular plants can be classified into hydraulic movements and movements caused by mechanical instabilities [18–19]. Given a sealing time of 3,300 s and a leaf diameter of 0.003 m as a basis, the sealing movements of *D. cooperi* leaves can be considered as mainly driven by hydraulic processes. However, additional processes driven by growth-induced mechanical pre-stresses in the tissues may also play an important role in this process [7].

**Figure 4:**
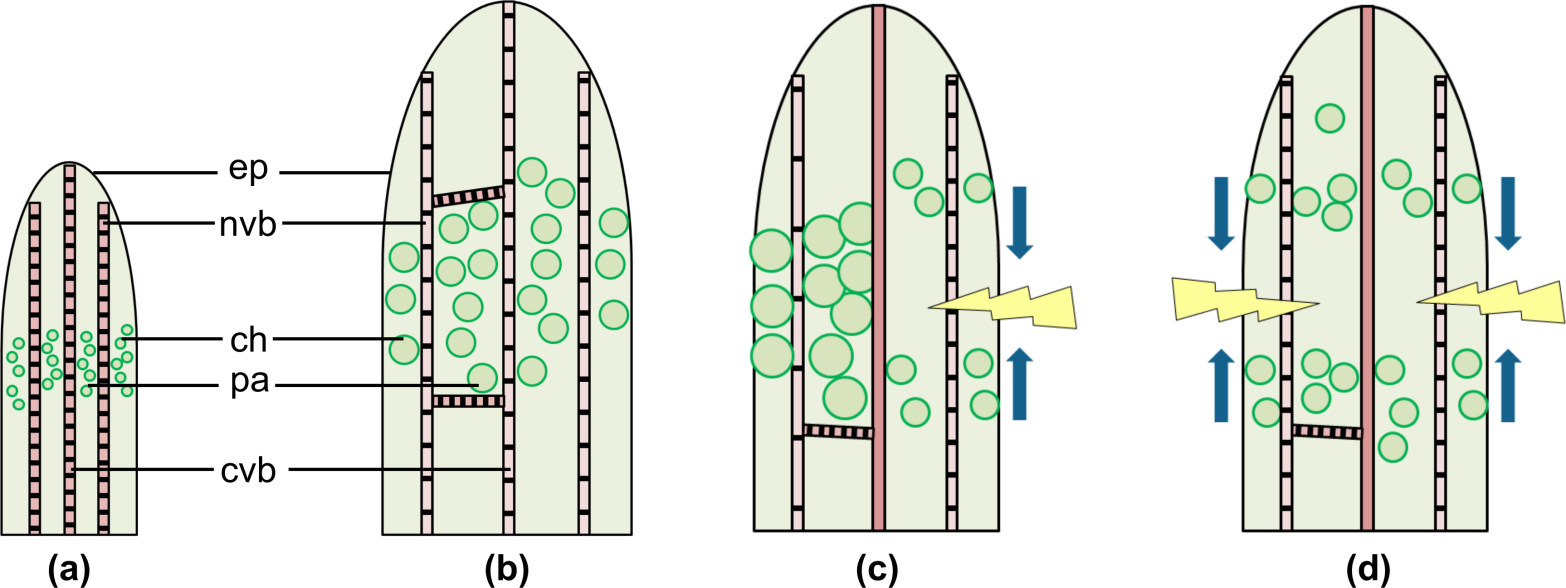
Schematic drawings of intact and damaged leaves. (a) Juvenile leaf, (b) adult leaf, (c) transversal or longitudinal cut, (d) ring incision. Illustration of self-sealing (c,d) represents low relative humidity conditions with the arrows indicating the closure of the fissure due to leaf bending (c) or leaf contraction (d). (ep) epidermis with window cells, (ch) chlorenchyma, (nvb) net of peripheral vascular bundles, (pa) parenchyma, (cvb) central vascular bundle. Examples of parenchymatous cells are illustrated.

We hypothesize that juvenile *D. cooperi* leaves contain small parenchymatous and chlorenchymatous cells and extensible vascular bundles with tracheids possessing annular and helical wall thickenings (Figure 4a). During ontogeny, the parenchyma and chlorenchyma cells take up increasing amounts of water, swell and become succulent (Figure 4b). As a consequence, in adult succulent leaves, the epidermis, the net of vascular bundles and the central vascular strand are pre-stressed under tension, whereas the parenchyma and chlorenchyma are pre-stressed under compression. In intact leaves, these internal pre-stresses provide the above-mentioned growth-induced and turgor-dependent overall equilibrium (Figure 4b).

The above-mentioned finding that the sealing movements of adult *D. cooperi* leaves fit well within the hydraulically driven range is supported by the humidity dependency of the relative bending angle (Figure 3). Our findings establish that the lower the relative air humidity, the higher the relative bending angle (closing of the fissure). Moreover, the observation that an added water droplet directly on the injury leads to negative values of bending angle (opening of the fissure) supports this interpretation (see Supporting Information File 2). Experiments in which a droplet of saturated salt solution was applied on the wound show strong osmotic effects. Previously turgescent cells in the wound region lose turgor pressure and become slack causing a downwards bending of the tip of the leaf according to gravity.

The dependency of the relative bending angle on size and velocity in relative humidity can be explained by the finding that, in a water-saturated atmosphere of 100% r.h., no or nearly no evaporation takes place in the chlorenchyma and parenchyma cells facing the fissure flanks and being exposed to the surrounding atmosphere. Therefore, their turgor, and consequently their cell size, remain (nearly) constant and no or only small and slow changes of the relative bending angle can be observed. In contrast, low relative air humidity increases evaporation and causes a (fast) reduction of turgor and size in the chlorenchyma and parenchyma cells facing the fissure flanks, resulting in marked and faster changes of the relative bending angle (Figure 3).

Several reasons might contribute to the significant differences of the self-repair efficiency found at 100% relative air humidity and after the direct application of a droplet of liquid water onto the fissure. First, different absolute amounts of water are present in the air (at 100% r.h.: 19.41 g/m³ of water) or in the droplet consisting of liquid water with small quantities of dissolved gas. Second, water accessibility is different, that is, the boundary layer energy is much higher in the case of water-saturated air (100% r.h.) than in the case of a macroscopic droplet of liquid water [20–21]. Third, liquid water from macroscopic droplets is sucked into the fissure by capillary and osmotic forces. This allows the hypothesis that the uptake of liquid water by the intact chlorenchyma and parenchyma cells results in a turgor-dependent increase of cell size and consequently an opening of the fissure.

The described turgor changes in chlorenchyma and parenchyma cells and the concomitant changes in tissue pressure and tissue stress are, in addition to the knowledge of the mechanical properties of the various tissue types comprising the *D. cooperi* leaf, important for the understanding of the supporting mechanical mechanism. This mechanism is hypothesized to increase the observed sealing movement and to improve the wound closure (Figure 3g). The effect is caused by the mechanical instabilities that come into play if fissures destroy the aforementioned mechanical equilibrium in a leaf that is composed of five tissue shells that are pre-stressed alternatively under compression or tension.

Konrad et al. [7] have developed an analytical elastic and visco-elastic model of intact and injured *D. cooperi* leaves exclusively taking into account mechanically driven mechanisms. Under certain conditions (e.g., depending on the cross-sectional tissue arrangement, elastic and visco-elastic properties of individual tissues and dimensions of the incision), the self-sealing mechanism has even been shown to work without hydraulic processes. Concerning internal stresses and strains, intact leaves are in an equilibrium state and store elastic energy that will be released in the case of a transversal, longitudinal or ring incision until a new mechanical equilibrium is established (Figure 4c, d). In the case of a transversal or longitudinal incision, the axially symmetric distribution of stresses within the leaf tissue is destroyed and finally results in a bending movement of the entire leaf (Figure 4c). First, a local pressure drop occurs on the cut side caused by the destruction of succulent parenchyma and chlorenchyma cells and the discharge of some water. On the opposite side, the pre-compressed parenchyma and chlorenchyma cells relax, an event that is not markedly impeded by the net of vascular bundles because of the extensibility of the wide-band tracheids forming this net. Second, the pretension of the wide-band tracheids in the net of vascular bundles results in a net tensile stress on the cut side of the leaf where no compressive counterbalance exists because of the destruction of the parenchyma and chlorenchyma cells [2]. The modelling by Konrad et al. [7] supports the idea that the new stress and strain distribution additionally increases leaf bending and improves the sealing of the incision. Leaf movement continues until a new equilibrium between the internal compressive and tensile stresses has been established. In the case of a ring incision, the axial symmetric distribution of stresses is preserved, although the general mechanical equilibrium is abolished (Figure 4d). First, a local pressure drop occurs in the wound region caused by the destruction of the precompressed parenchymatous and chlorenchyma cells and the discharge of some water. Second, observations suggest that the contraction of the pretensioned net of wide-band tracheids causes a net stress, pulling the apical part of the leaf towards the leaf base. As long as overall tensile stresses dominate, a contraction of the leaf takes place until a new equilibrium between the internal compressive and tensile stresses has been established [2,7]. The dimensions and mechanical properties measured or calculated for the five different tissue types found in *D. cooperi* leaves also corroborate the results of modelling the existence of such a supporting mechanically driven mechanism.

Subsequent to the relatively fast initial self-sealing processes, self-healing including biosynthesis and cell proliferation starts, and hence the formation of a specific wound healing tissue. After a few weeks, self-healing is completed showing pronounced healed tissue in the wound region. In some cases, the leaves remain in a curved shape because of a previous lateral injury (Figure 1b). Our studies show that even deep injuries can be healed and that the apical part of the leaf above the injury remains intact.

## Experimental

### Plants

*Delosperma cooperi* (Hook f.) L. Bolus (Figure 1) is a perennial plant belonging to the Aizoaceae family. Forming dense lawns in its natural habitats, the plants reach a height of approximately 20–40 cm, with fleshy leaves and trailing stems. *D. cooperi* grows in the cold semi-arid climate of Eastern Cape (South Africa) and Free State at altitudes between 1,350 and 2,745 m above sea level [16–17]. Test plants were obtained from greenhouse cultivations in the Botanic Garden of the University of Freiburg (Germany). Adult plants were cultivated under the following growth conditions: temperature 18.3 ± 3.3 °C and relative humidity 66.4 ± 6.0%. Temperature and relative humidity were measured by using the data logger Testo 175-H2 38237162 (Testo SE & Co. KGaA, Lenzkirch, Germany).

### Anatomy

Nawaschin’s fluid (mixture of equal portions of solutions A and B; A: 5 mL 10% chromic oxide, 3.5 mL acetic acid, 42 mL distilled water; B: 15 mL 37% formaldehyde, 35 mL distilled water) was used for the fixation of leaf samples. After dehydration with increasing ethanol concentrations, the samples were embedded in 2-hydroxymethacrylate (Technovit 7100, Kulzer, Wehrheim/Ts, Germany) according to the manufacturer’s instructions. A custom-built rotary microtome (Technical Workshop, Institute of Biology II/III, University of Freiburg, Germany) was used to cut thin sections between 5 µm and 10 µm in thickness. Overview staining with toluidine blue (0.05% toluidine blue in distilled water) was used to give contrast to the various leaf tissues. Unlignified primary cell walls appeared dark blue and lignified secondary cell walls were light blue. Permanent slides were prepared by using the mounting medium Entellan (Merck KGaA). Sections were examined with an Olympus BX61 microscope (Olympus Corporation, Tokyo, Japan) equipped with a DP71 camera module. Parameters of the cells and tissues were determined with the image analysis software ImageJ 1.46h.

### Biomechanics

#### Elastic moduli of leaves, epidermis and central vascular bundle

The biomechanical properties of entire leaves and of single tissue layers (epidermis, central strand of vascular bundles) were studied in tensile tests performed on a modified custom-made micro-tensile-testing device (Technical Workshop, Institute of Biology II/III, University of Freiburg, Germany, for details see [22]). The device was equipped with microstep motors with an accuracy of ±3 µm, a high precision linear table, a compression–tension load cell with a maximum load of 10 N and a resolution of ±10 mN (model 31E, Honeywell, Columbus, OH, USA) and two sample holders (aluminium platelets) on opposite sides. The platelets were fitted with holes for easy mounting onto the tensile apparatus via a pinhole assembly. Experimental control and the reading of data were performed by a measurement amplifier (Spider 8, Hottinger Baldwin Messtechnik GmbH).

The ends of the samples were stuck to the sample holders with a rapid cyanoacrylate adhesive (Uhu Sekundenkleber blitzschnell Pipette, UHU GmbH and Co. KG Bühl, Germany). The samples were carefully arranged parallel to the tension forces to ensure an even strain field over the diameter of the sample. During hardening the adhesive glued samples were stored in a humidity chamber (>95% relative air humidity) in order to prevent or slow down dehydration (storage time for leaves: 2–4 h, storage time for strands of vascular bundles and epidermis samples: a few minutes). After glue hardening, the platelets were mounted onto the tensile apparatus and the displacement tests were conducted. During the tensile tests, images were taken at a frame rate of one image per second by use of an Olympus SZX9 dissecting microscope via a Color View II digital camera (Soft Imaging Systems GmbH, Münster, Germany) and the software cell D. Measured values of time *t*, force *F* and displacement Δ*L* were captured at five readings per second. The strain rate was 0.002 s^−1^.

During the testing of whole leaves, images were taken from two sides in order to allow the measurement of the original length *L*_0_, height (2*b*) and width (2*a*). The height and width were used to calculate the cross-sectional area. Before the start of the tensile tests, the leaves were marked with a black line at mid-length to ensure that transverse strains and axial strains were always calculated from the same point for the subsequent calculation of Poisson’s ratio. For the testing of isolated tissues, such as central vascular bundles or epidermis, adjacent tissues were removed carefully and as completely as possible. Thin-sections were prepared from epidermis samples and from the basal and apical region of the tapered strands and cross-sectional areas were calculated. The epidermis was tested in a longitudinal and in a transversal direction to test possible anisotropy in this tissue layer. To reduce dehydration effects during tensile tests, epidermis samples and strands of vascular bundles were sprayed with finely dispersed water vapour from an ultrasound nebuliser. The images were taken with an Olympus SZX9 dissecting microscope via a Color View II digital camera (Soft Imaging Systems GmbH, Münster, Germany) and the software cell D. The measurements were conducted with the software ImageJ 1.46h.

The recorded force–displacement data of the tested leaves and tissues were used to assess the tensile strength and elastic modulus. On the basis of the images taken, the height (2*b*) and width (2*a*) of each leaf were measured at five points along the longitudinal axis of the leaf. Every cross-sectional area (*A*_leaf_) was calculated (Equation 2) assuming an elliptical shape of the leaf; this holds true as a good approximation in this species.

[2]
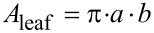


For further calculations, the mean cross-sectional area (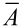) of the leaf was determined as the mean value of the five calculated cross-sectional areas along each leaf. The cross-sectional area of the tapered strands of vascular bundles was calculated from the basal radius achieved from thin sections. Because of the large diameter of the window cells, the cross-sectional area of the epidermis was difficult to assess. The height and width were determined based on a rectangle, from thin sections.

Stress (σ) was calculated (Equation 3) as the force (*F*) per mean cross-sectional area (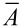) of a tested sample.

[3]
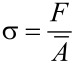


The tensile strength (σ_max_) is given by (Equation 4) the maximum force (*F*_max_) per mean cross-sectional area (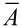).

[4]
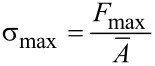


The strain (ε) was calculated (Equation 5) as the fraction of the displacement (Δ*L*) divided by the original length (*L*_0_) of a tested sample.

[5]
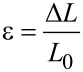


The elastic modulus, a measure of material stiffness, was calculated (Equation 6) from the slope of the initial (in good approximation) linear (i.e., elastic) part of the respective stress–strain curve (σ/ε).

[6]
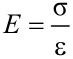


The Poisson’s ratio (ν) of a leaf is calculated as the quotient (Equation 7) of transverse strain (ε_trans_) and axial strain (ε_axial_), where *d* = 2*a*. The first image taken in the linear-elastic range was used for measuring *L*_0_ and *d*_0_. The linear-elastic range Δ*L* and Δ*d* were determined from the last image taken.

[7]
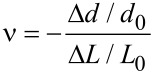


#### Elastic moduli of parenchyma and chlorenchyma

The linear relationship between the elastic modulus of the parenchyma or chlorenchyma and turgor can be described by Equation 1:

[1]
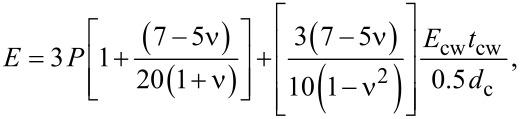


where *P* is the turgor, *d*_c_ is the cell diameter, *t*_cw_ is the cell wall thickness, ν is Poisson’s ratio and *E*_cw_ is the elastic modulus of the cell wall [23]. The first term describes the contribution of turgor to the elastic modulus of the tissue, whereas the second term of Equation 1 expresses the influence of the geometry and mechanical properties of cells and cell walls.

The turgor of single parenchyma and chlorenchyma cells was measured at various positions on the leaf by using a cell pressure probe (Lehner GmbH Sensor Systeme, Kirchheim/Teck, Germany). The cell pressure probe was equipped with a 0 to 1.6 MPa pressure transmitter (DRT-AL-20MA-R16B, Hygrosens Instruments GmbH, Löffingen, Germany), microstep motors for propulsion and an internal indenter. The measurements were conducted according to the procedure proposed by Thomas et al. [24]. Microcapillaries were pulled with a horizontal micropipette puller (Narishige PD-5, Tokyo, Japan). Tips were broken by stabbing them through paper wipes and were selected under a microscope for clean edges and small tip diameters (tips with a mean tip diameter of 5–10 µm); these selected tips were used for the measurements. For the optical control of the oil/cell sap meniscus, we used a Zeiss universal microscope with incident illuminator (Zeiss H-PL-Pol) and a custom-built specimen holder with micromanipulators [25]. The measurement of cell turgor of the parenchyma and chlorenchyma was carried out on adult leaves of undamaged plants in pots.

### Self-sealing efficiency

**Figure 5:**
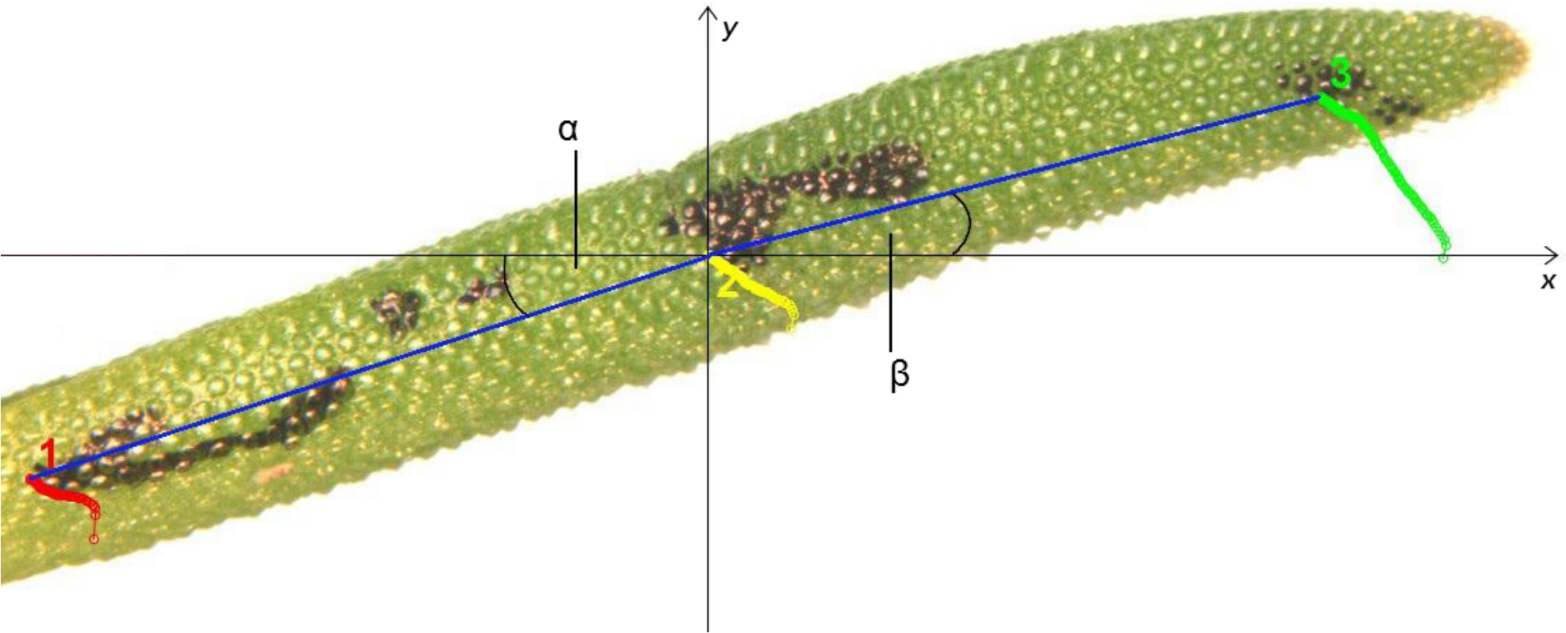
The evaluation of self-sealing. The tracking of three defined points at the leaf base (1), close to the injury (2) and at the leaf tip (3) during the bending of the injured leaf is the basis for calculating the angles α and β. Further calculations lead to the relative bending angle γ, which represents a quantitative measure for the self-sealing movement during a given time span.

The measurement of self-sealing efficiency in terms of the relative bending angle γ was carried out on leaves of potted plants. The upper side of the leaf was injured in a transversal direction by a razor blade. The cutting depth was chosen so that in each experiment, the parenchyma was injured with certainty. On average, the parenchyma starts at 55% of the radius relative to the leaf centre [7]. Beginning with an image of the undamaged leaf and then at every 30 sec after the injury, an image was taken with an Olympus SZX9 dissecting microscope via a Color View II digital camera (Soft Imaging Systems GmbH, Münster, Germany) and the software cell D. The images were recorded over a period of 55 min. The movement of the leaf was determined by measuring three points on the leaf (Figure 5). The first point was selected in the vicinity of the leaf base, the second point close to the injury, and the third one at the leaf tip. The movement of the three points was analysed with the software ImageJ 1.46h and the aid of the Plugin MTrackJ.

In each image, the second point was chosen to be the origin of the coordinate system. Starting from this zero point, the two angles α and β were calculated relative to a horizontal line and the straight lines between points 2 and 1 and between points 2 and 3, respectively. The variations in the angle α represent the relative movement of the leaf base whereas variations in angle β were a measure of the rotation of the leaf tip with respect to point 2. The additional movement of the leaf base could be eliminated by the subtraction of angle α from angle β. This led to the actual bending angle δ, representing the actual closing movement of a leaf after an injury. The relative bending angle *γ*_n_ was calculated from the difference of the actual bending angle (δ_n_) at time *t* = *n* min and the actual bending angle before injury, that is, at the beginning of the movement (δ_0_) at time *t* = 0 min. Positive values of the relative bending angle represented a closure of the lesion, whereas negative values indicated an opening of the wound (Supporting Information File 1 and Supporting Information File 2). The experiments were carried out at 24, 33, 49 and 100% relative air humidity and after a water droplet was applied on the wound within times spans of 1, 5, 10, 20, 40 and 55 minutes.

The temperature and relative humidity were measured by using a data logger 175-H2 (Testo, Lenzkirch, Germany). At a temperature of 22.95 ± 2.26 °C, the absolute amount of water vapour in the air varies with the humidity as follows: 24% = 4.66 g/m³, 33% = 6.40 g/m³, 49% = 9.51 g/m³, and 100% = 19.41 g/m³.

### Statistics

The datasets were analysed by using the statistical software GNU R 3.2.3 [26]. The majority of tests were conducted by using built-in functions of the software R; however, some functions of the packages car [27], psych [28] and ez [29] were also used in our statistical approaches. Normally distributed tissue characteristics were described by using the mean ± standard deviation (SD), whereas the non-normally distributed mechanical leaf characteristics were described by median and interquartile ranges (IQR).

The relationship between the turgor pressure of single cells and their position in the leaf was analysed by using Spearman’s rank order correlation. Finally, the influence of “humidity conditions in the wound region” and “time after wounding” on the relative bending angle was analysed by using a two-way repeated measure ANOVA on rank-transformed data. Post-hoc tests were executed either analytically for the single main effects (pairwise comparisons by using Student’s *t*-tests and Bonferroni *p*-value adjustments) or graphically for their interaction effect. All required assumptions were met. The normality of residuals was tested graphically by using QQ-plots. Mauchly’s test for sphericity together with the Greenhouse–Geisser correction method was used to evaluate sphericity and to account for possible deviations.

## Supporting Information

File 1 Leaf of *Delosperma cooperi*, transversal cut, real time 55 min, ca. 20% r.h.

File 2 Leaf of *Delosperma cooperi*, transversal cut, real time 55 min, water droplet.

File 3 Additional statistical analysis.
Statistical analysis concerning the influence of “humidity conditions in the wound region” (relative air humidity and water droplet) and “time after wounding” on the relative bending angle.

File 4 Raw data.
