# Supplementary material for: Humidity-dependent wound sealing in succulent leaves of Delosperma cooperi – An adaptation to seasonal drought stress
Source: Beilstein J Nanotechnol. 2018 Jan 16;9:175–86. doi: 10.3762/bjnano.9.20 (PMC5789399; doi:10.3762/bjnano.9.20)
Supplement: File 3 — Additional statistical analysis. Statistical analysis concerning the influence of “humidity conditions in the wound region” (relative air humidity and water droplet) and “time after wounding” on the relative bending angle. [file Beilstein_J_Nanotechnol-09-175-s003.pdf]

Supporting Information

for

Humidity-dependent wound sealing in succulent leaves of *Delosperma cooperi* – An adaptation to seasonal drought stress

Olga Speck*[1,2,3], Mark Schlechtendahl[1,4], Florian Borm[1], Tim Kampowski[1,4], and Thomas Speck[1,2,3,4]

Address: [1]Plant Biomechanics Group, Botanic Garden, Faculty of Biology, University of Freiburg, Schänzlestraße 1, 79104 Freiburg, Germany, [2]Competence Network Biomimetics, Baden-Württemberg, Schänzlestraße 1, 79104 Freiburg, Germany, [3]Freiburg Center for Interactive Materials and Bioinspired Technologies (FIT), University of Freiburg, Georges-Köhler-Allee 105, 79110 Freiburg, Germany, and [4]Freiburg Materials Research Center (FMF), University of Freiburg, Stefan-Meier-Str. 21, 79104 Freiburg, Germany

Email: Olga Speck - olga.speck@biologie.uni-freiburg.de

* Corresponding author

Additional statistical analysis

Statistical analysis concerning the influence of "humidity conditions in the wound region" (relative air humidity and water droplet) and "time after wounding" on the relative bending angle.

**Two-way repeated measures ANOVA on rank-transformed data***					
	Df	SS	MS	*F*	*p*-value
"Humidity conditions in the wound region"	3	3187050	1062350	73.31	$p < 0.001$
"Time after wounding"	5	265535	53107	29.86	$p < 0.001$
"Humidity conditions in the wound region" vs "Time after wounding"	15	766988	51133	40.51	$p < 0.001$

**Post-hoc testing: pairwise comparisons using Student's *t*-tests****		
"Humidity conditions in the wound region"	24% r.h. vs 49% r.h.	$p < 0.001$
	24% r.h. vs 100% r.h.	$p < 0.001$
	24% r.h. vs water drop	$p < 0.001$
	49% r.h. vs 100% r.h.	$p < 0.001$
	49% r.h. vs water drop	$p < 0.001$
	100% r.h. vs water drop	$p < 0.001$
"Time after wounding"	1 min vs 5 min	$p < 0.001$
	1 min vs 10 min	$p < 0.001$
	1 min vs 20 min	$p < 0.001$
	1 min vs 40 min	$p < 0.001$
	1 min vs 55 min	$p < 0.01$
	5 min vs 10 min	$p < 0.01$
	5 min vs 20 min	$p < 0.05$
	5 min vs 40 min	$p > 0.05$
	5 min vs 55 min	$p > 0.05$
	10 min vs 20 min	$p > 0.05$
	10 min vs 40 min	$p > 0.05$
	10 min vs 55 min	$p > 0.05$
	20 min vs 40 min	$p > 0.05$
	20 min vs 55 min	$p > 0.05$
	40 min vs 55 min	$p > 0.05$

*A two-way repeated measures ANOVA on rank-transformed data was performed. The degrees of freedom (Df), sums of squares (SS), mean squares (MS), *F*-statistics and *p*-values of both the single main effects ('humidity conditions in the wound region' and 'time after wounding') and their interaction effect are presented.

**For post-hoc testing, pairwise comparisons using Student's *t*-tests and Bonferroni *p*-value adjustments were carried out in case of the single main effects. A graphic representation was used to post-hoc test their interaction effect (see Figure 3g). The required assumptions (normality of residuals and sphericity) have been met.
